# Analgesia in conjunction with normalisation of thermal sensation following deep brain stimulation for central post-stroke pain

**DOI:** 10.1016/j.pain.2009.09.011

**Published:** 2009-12-15

**Authors:** Anthony E. Pickering, Simon R. Thornton, Sarah J. Love-Jones, Charlotte Steeds, Nikunj K. Patel

**Affiliations:** a Department of Physiology and Pharmacology, University of Bristol, UK; b Department of Anaesthesia, North Bristol NHS Trust, UK; c Department of Neurosurgery, North Bristol NHS Trust, UK; d Department of Anaesthesia, University Hospitals Bristol, UK

**Keywords:** Deep brain stimulation, Central post-stroke pain, Thalamic dysrhythmia, Allodynia

## Abstract

The aetiology of central post-stroke pain (CPSP) is poorly understood and such pains are often refractory to treatment. We report the case of a 56-year-old man, who, following a temporo-parietal infarct, suffered from debilitating and refractory hemi-body cold dysaesthesia and severe tactile allodynia. This was associated with thermal and tactile hypoaesthesia and hypoalgesia on his affected side. Implantation of a deep brain stimulating electrode in his periventricular gray (PVG) region produced an improvement in his pain that was associated with a striking normalisation of his deficits in somatosensory perception. This improvement in pain and thermal sensibility was reversed as stimulation became less effective, because of increased electrode impedance. Therefore, we postulate that the analgesic benefit may have occurred as a consequence of the normalisation of somatosensory function and we discuss these findings in relation to the theories of central pain generation and the potential to engage useful plasticity in central circuits.

## 1 Introduction

Some of the landmark clinical reports of chronic pain following cerebrovascular events appeared at the turn of the last century [15,20] however central post-stroke pain (CPSP) remains a poorly understood condition that is often refractory to treatment [6]. It is now appreciated that CPSP is a relatively common sequela of cerebrovascular accidents (CVA), developing in around 8% of patients [4]. The disease burden of CPSP is increasing as the prevalence of stroke rises in our ageing population and it has been estimated that around 28,000 people suffer from this condition in the UK [34]. CPSP is characteristically severe, continuous and often has a hemi-body distribution. Patients report altered thermal sensation (burning or freezing) and tactile or cold allodynia [25]. Sensory examination often reveals an apparently paradoxical deficiency of warm, cold and touch sensibility in the regions of thermal and tactile allodynia [7,8].

CPSP may occur secondary to lesions of a range of brain structures along the neuraxis although it is most commonly associated with lesions of the sensory thalamus (which can be very discrete [24]). The unifying pathology seems to be interruption of the transmission of information in the spino-thalamo-cortical tracts which convey thermal and pain sensibilities from the periphery (reviewed in [6]). This, in part, may account for the association between CPSP and thermosensory abnormalities. Several potential neural mechanisms have been proposed to account for the puzzling cluster of signs and symptoms of CPSP [8,11,12,29] but as yet none of these theories have been directly testable in man nor have they led to therapeutic advances as the pathology has appeared secondary to an irreversible loss of neural function.

Because CPSP patients are poorly responsive to both conventional analgesic therapies and to treatments targeted against neuropathic pains, some groups have examined the role of functional neurosurgery and central neuromodulation. In particular recent studies of deep brain stimulation (DBS) and motor cortex stimulation have reported some beneficial effects in refractory CPSP patients [23,34]. Here we report the case of a 56-year-old man, who, following a right-sided temporo-parietal infarct, presented with typical symptoms of CPSP. Quantitative sensory testing prior to implantation of DBS electrode showed thermal and tactile hypoaesthesia and hypoalgesia on his affected side. After DBS of the periventricular gray (PVG) region he showed a marked improvement in his pain that was associated with a normalisation of the deficits in somatosensory perception. This improvement in pain and thermal sensibility was reversed as stimulation became less effective, because of increased electrode impedance. Therefore we suggest that the analgesic benefit may have occurred as a consequence of the normalisation of somatosensory function and we discuss these findings in relation to the theories of central pain generation and the potential to engage useful plasticity.

## 2 Case report

### 2.1 History and examination

ML, a 56-year-old gentleman, initially presented at the age of 51, with a one day history of headache followed by the sudden onset of dense left-sided weakness. His computerised tomography scan on admission showed “*an area of poorly defined low density related to the right internal capsule, which most likely represents recent infarction*”. He was a smoker and his past medical history included longstanding back pain with sciatica secondary to disc prolapse (which had forced medical retirement from work). While an inpatient he was diagnosed with, and treated for, hypertension and hypercholesterolemia.

His initial left hemiparesis resolved over 6 weeks but was gradually replaced (from week 3 onwards) by left hemi-body pain (rated as 10/10 every day). He described having “frostbite” on the whole of the left side of his body and such severe tactile allodynia that he found clothing difficult to tolerate. A diagnosis of central pain syndrome was made and, following referral to a chronic pain clinic, he was given trials of nortriptyline (60 mg nocte), gabapentin (600 mg three times daily), nabilone, acupuncture and physiotherapy. His pain was refractory to these treatments and consequently he was referred to a tertiary pain clinic to assess his suitability for neuromodulation.

At this point, 2 years after his CVA, he described an ‘ice cold’, dull pain down the left hand side of his body which was worst in his lower leg and foot. His symptoms were exacerbated by cold and wet weather. Tactile allodynia was still a prominent feature and he complained of consequent difficulties with normal human contact. He obtained little benefit from further trials of combinations of strong opiates, pregabalin and tricylic antidepressants. TENS also failed to provide symptomatic relief. Therefore, a trial of spinal cord stimulation (SCS) was undertaken with the lead placed at the T11/12 level with the aim of alleviating the pain in his leg (his worst pain). However, despite being technically successful (evoking paraesthesia in the leg) SCS failed to improve his pain and was abandoned. Against this background, and after psychological assessment for suitability, ML was scheduled for DBS.

Prior to DBS implantation ML completed baseline pain questionnaires: Brief Pain Inventory (BPI) [9] and Neuropathy pain scale (NPS) [17]. We also performed a formal neurological examination and quantitative sensory testing (QST). He rated his average pain as 9/10 and his mean pain interference score on BPI was 7.7/10. He had an overall NPS score of 82/100 (see Table 1 for component scores).

**Fig. 1:**
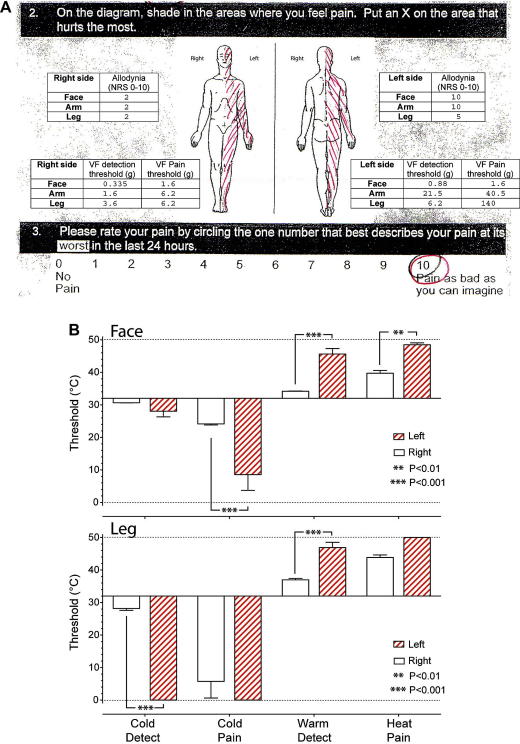
Baseline pain assessment. (A) Extract from original BPI assessment tool showing hemi-body distribution of pain and score of 10/10 (NRS) for the worst pain in last 24 h. Overlaid are the results of tactile QST showing profound dynamic allodynia on cotton bud stroke on the left side and an associated insensitivity to static tactile stimuli with elevated thresholds for detection and pain (assessed with von-Frey hairs). (B) Assessment of thermal detection and pain thresholds showed significant elevations of both cold and warm detection thresholds on the left side (face and leg) and also an increase in the heat and cold (face) pain thresholds indicating the presence of left-sided thermal hypoaesthesia and hypoalgesia (Students *t*-test).

ML had considerable difficulty disrobing because of his hemi-body allodynia and he forcefully declined a formal peripheral neurological examination of his left upper and lower limbs. Indeed he flinched and attempted to withdraw whenever contact was impending or his personal space was encroached on his affected side. Tone, power, reflexes and coordination were all normal on the unaffected side. Cranial nerve examination was unremarkable other than left facial tactile allodynia. ML agreed to have QST on the anterior shin, volar forearm and maxillary areas bilaterally. Brushing with a cotton bud evoked severe allodynia (10/10 NRS on face and upper limb) on the left side and mild allodynia on the right (2/10 NRS). Punctate mechanical sensation was assessed using von-Frey hairs, showing left-sided hypoaesthesia and hypoalgesia (Fig. 1A). Assessment of thermal sensation (TSA II™ thermosensory analyser, Medoc, Israel) demonstrated marked warm and cool hypoaesthesia and hot and cold hypoalgesia on his left side (Fig. 1B).

### 2.2 Surgical procedure

**Fig. 2:**
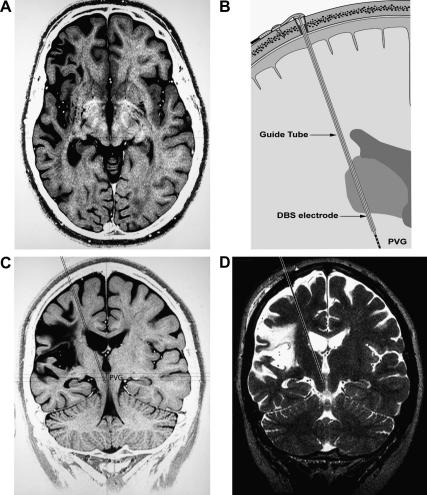
DBS system implantation. (A) Axial planning MRI scan (inverted T2 weighted) showing atrophic area of infarct territory in right fronto-parietal cortex (including insula) extending to internal capsule (3 years after the original infarct). (B) Schematic of implant showing the guide-tube containing the DBS lead inserted to target. (C) Coronal MRI planning view (inverted T2 weighted) showing the target area in the periventricular gray (PVG) and the intended trans-ventricular electrode track (dotted lines). (D) Peri-operative MRI (T2 weighted) showing position of the stylette (dotted lines) in the target region.

Our methods for magnetic resonance imaging guided placement of DBS electrodes have been described by us in detail previously [35] and are only presented here in outline (shown schematically in Fig. 2B).

#### 2.2.1 Procedure 1

Under general anaesthesia a modified MRI compatible Leksell frame was applied and detailed MRI scans were performed which showed the extent of the infarct damage to cortical territories extending from the superior temporal gyrus through the supra-marginal gyrus to the superior parietal lobule and including the insula. Below the cortex there was damage to the posterior external and internal capsules and to the ventrolateral thalamus. A trajectory was planned to target the PVG (based on previous reports of benefit in CPSP [34]) with a trans-ventricular approach (Fig. 2A and C). This route was chosen to optimise the axial placement of the stimulating electrode in the PVG/PAG thus maximising the likelihood of finding an appropriate stimulus location within this midline territory.

#### 2.2.2 Procedure 2

The following day the patient was re-anaesthetised and a right frontal burrhole was made along the planned trajectory. Stereotaxic placement of a probe allowed the guide-tube to be railroaded into position 12 mm from the final target. The probe was replaced by a radio-opaque stylette to target. The correct positioning in the right periventricular and periaqueductal gray was confirmed on repeat MRI scan (Fig. 2D) whereupon the stylette was removed and replaced with a DBS lead (Medtronic 3387). This was tunnelled subcutaneously and connected to a pectoral Kinetra generator (Medtronic).

### 2.3 Post-operative course

After optimisation of the stimulation parameters (pulse amplitude 2 V, duration 240 μs at 5 Hz) ML’s pain was considerably reduced along with a marked improvement in his allodynia. It was noted, during optimisation, that there was a degree of somatotopy evident on stimulation with the most proximal (apical) electrodes affecting sensation from the lower limbs and the caudal electrodes affecting the face/upper limbs (as previously noted [5]). It was also apparent that the beneficial effects of stimulation were reversed (within minutes) if stimulation ceased with pain rapidly returning to its previous severity.

Some 6 weeks post DBS implantation ML was followed up in clinic. He had a qualitative symptomatic improvement (pain score reduced from 10/10 to 4/10 NRS), better affect and little sign of fear of personal contact. There had been no change in his analgesic medication during this period (oxycodone and gabapentin).

**Fig. 3:**
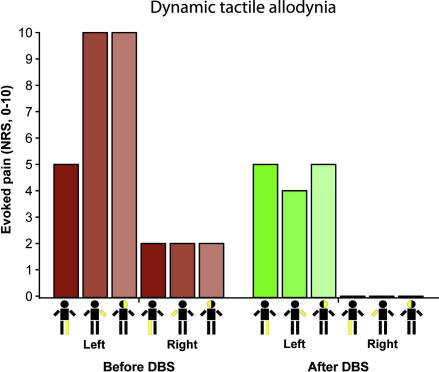
DBS attenuates dynamic tactile allodynia. Brush strokes with a cotton bud (2–3 cm/s over a 6 cm distance) evoked severe tactile allodynia down his left side (worst in arm and face 10/10 NRS) with milder allodynia evoked on the right. Six weeks after PVG DBS there was a clear improvement in allodynia with around a 50% reduction on the affected side and a complete resolution on the right.

**Fig. 4:**
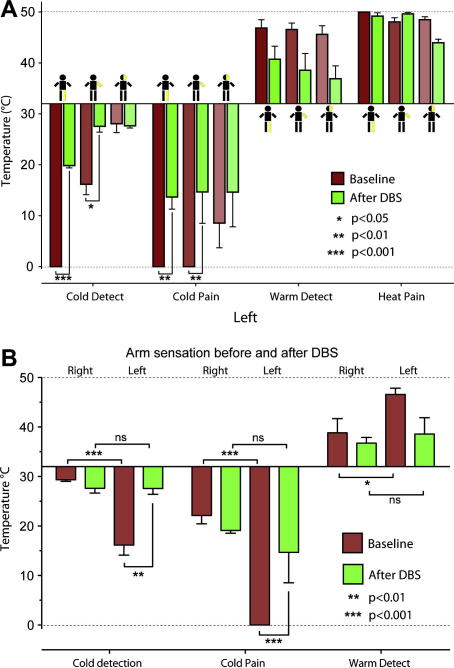
Resolution of thermal sensory abnormalities with DBS. (A) QST of affected left side (medial shin, volar forearm and maxilla) before and after DBS Showed the presence of cold and warm hypoaesthesia and cold hypoalgesia. After PVG DBS there was a significant improvement in the cold detection and cold pain thresholds with a trend also seen towards normalisation of the warm detection threshold. (B) Comparison of the left and right upper limbs before and after DBS. The significant side-to-side differences in thermal detection and cold pain thresholds were resolved by PVG DBS (repeated measures ANOVA with Bonferoni post tests ∗*p* < 0.05, ∗∗*p* < 0.01, ∗∗∗*p* < 0.001).

We again administered the pain questionnaires and repeated QST. His NPS had reduced from 82 to 49 with a complete resolution of the deep pain and improvements in the other aspects of 30–50% (Table 1). He estimated the degree of pain relief provided by DBS as 70% and showed improvements in his BPI interference scores. QST showed that his allodynia had improved considerably in the arm and face (NRS 4 and 5/10 respectively, Fig. 3) on the affected side and the mild right-sided allodynia had completely resolved. His tactile hypoalgesia and hypoaesthesia had also improved on the affected side, for example his left arm detection threshold improved from 21 to 0.6 g and the pricking threshold from 40.5 to 6.2 g. Equally striking was the change in thermal thresholds (Fig. 4A) with almost complete resolution of the previous left-sided hypoalgesia and hypoaesthesia without any significant corresponding change in the thresholds on the right (Fig. 4B).

ML was reviewed in clinic, some 4 months after implantation, because of a gradual deterioration in his pain control. He stated that his pain had returned towards pre-operative levels and this was borne out by his BPI assessment which showed his average pain as 10/10 NRS with an average interference score of 9.4/10 NRS. Interrogation of his DBS system revealed that the impedance across the contacts of the stimulating electrode had almost doubled and the stimulation parameters needed to be adjusted. Repeat QST at this point (prior to DBS adjustment) showed that the left-sided thermal (but not punctate mechanical) hypoalgesia and hypoaesthesia had returned along with the evoked dynamic allodynia (7–8/10). After adjustment of his DBS parameters (increased pulse amplitude), a significant improvement in both his pain and allodynia was achieved (he declined further QST after this adjustment). Over the following year the benefit from DBS was not maintained and there was an increase in his pain scores. However, he was unwilling to turn the stimulator off as he found this worsened his pain, suggesting the stimulation was still producing some analgesic effect.

## 3 Discussion

We have presented a patient with a severe, refractory central pain syndrome following a temporo-parietal infarct manifesting as hemi-body pain with cold dysaesthesia and marked tactile allodynia. This was associated with thermal hypoaesthesia and hypoalgesia along with decreased punctate tactile sensation. DBS of the periventricular gray produced a striking analgesic effect with reduced allodynia that was associated with resolution of the hemi-body hypoaesthesia and hypolagesia. Unfortunately, this improvement lasted less than 9 months and as his pain returned so his sensory loss recurred. This is therefore the first report of therapeutic restoration of sensory function associated with an improvement in CPSP symptoms.

Head and Holmes [20] suggested central pain was caused by loss of specific pain and temperature pathways as a result of damage to the lateral thalamus disinhibiting the medial thalamic nucleus. This hypothesis has been re-formulated to postulate that the loss of input in the neo-spinothalamic tract removes the sensori-discriminative aspects of pain input leaving the phylogenetically older medial pain pathways intact and without their usual regulation [8].

A further refinement of the disinhibition theory suggests that it is the loss of normal cool sensory input from the periphery that removes a tonic inhibitory influence on thalamic wide-dynamic range neurones giving rise to the sensation of burning pain and allodynia [11,12]. Craig has also implicated the insula as having a role in the generation of CPSP [13]. This is consistent with the observation that parietal cortex lesions involving the insula can produce CPSP [40] with similar symptomatology as that exhibited by ML. However, QST in a series of CPSP patients has not shown the predicted close association between cold hypoaesthesia and cold allodynia [18] suggesting that this hypothesis alone does not account for the pain in all subjects.

All of these preceding hypotheses emphasise the role of anatomical damage to sensory pathways as being the fundamental mechanism responsible for the generation of CPSP. There have also been suggestions of functional deficits; for example it has been proposed that there are alterations within the reticular nucleus of the thalamus leading to an atypical oscillating pattern of neural activity and thence to altered sensory transmission through the thalamus [29]. Consistent with this idea is the observation of abnormal excitability of thalamic units in patients with deafferentation pains [39]. A similar observation has been made in patients with CPSP who have abnormal oscillatory thalamic field potentials at 0.2–0.4 Hz that were attenuated by PVG DBS [32]. These findings suggest CPSP may be a consequence of a functional thalamic dysrhythmia as proposed by Llinas et al. [26,27]. It is worthy of note that thalamic dysrhythmias have also been implicated in the pathology of other neurological disorders such as Parkinson’s disease, and it has been suggested that the beneficial effects of DBS in Parkinson’s disease may be due to an improvement in such dysrhythmias [27].

Stimulation of deep brain structures has been used as a therapy in a variety of forms for over 40 years [2,22,38] and has targeted a range of structures including thalamic nuclei [31] and the periaqueductal and periventricular gray (PAG and PVG) [21]. Although CPSP was originally considered to be poorly responsive to DBS, some recent reports have indicated that DBS may be of benefit for some patients [23,34]. Owen et al. [34] found better results in the treatment of CPSP when stimulating the periventricular gray region as compared to thalamus and we found a similar effect in our case. However, no previous DBS study has ever noted an improvement in sensory function associated with the analgesic benefit in CPSP (or indeed any other neuropathic pain condition). Intriguingly, there has been a report of an improvement in motor symptoms (upper limb paresis) by PVG DBS for CPSP in a patient with a posterior cerebral artery territory infarct [36].

The initial hypothesis underpinning the introduction of DBS was based on dramatic animal studies that showed electrical stimulation of the periaqueductal gray (PAG) evoked profound analgesia [30,37]. These animal studies went onto suggest that this analgesia was, at least in part, a result of activation of an endogenous opioid system [3] thought to involve a descending relay in the medulla to alter nociception at the level of the spinal cord. There is evidence supporting a role of endogenous opioids in the mediation of the analgesic effect of DBS in patients [1,21,42] but there are also non-opioidergic mechanisms (reviewed by Duncan et al. [16]).

It would seem unlikely that release of endogenous opiates alone could account for the improvements in sensory function noted to accompany the analgesic benefit seen in ML. Rather, the release of endogenous opioids would have been expected to increase pain thresholds (infusion of opioids increases heat pain threshold without effect on cold pain threshold [19]). It is noteworthy that there are ascending connections from the PAG to the thalamus in primates [28]. Such projections have previously been proposed to be responsible for the damping action of PVG DBS on the aberrant thalamic activity seen in CPSP [32]. If such thalamic dysrhythmia is indeed responsible for the generation of both the positive (allodynia and spontaneous pain) and negative features (hypoalgesia and hypoaesthesia) of CPSP, as has been proposed [26,27], then this could account for the ability of DBS to reversibly improve apparently “hard wired” neurological deficits in ML. Alterations in thalamic function have also been suggested to underlie some of the beneficial effects seen with motor cortex stimulation in CPSP [33,41].

Although these findings are encouraging, unfortunately the analgesic benefits of DBS were relatively short-lived in the case of ML, due to increased electrode impedance (perhaps secondary to local gliosis) which limited the effectiveness of stimulation. It is also possible that ML developed stimulation tolerance as has been seen with DBS for other chronic pains (reviewed in [10]). The limited evidence base supporting the use of DBS in CPSP has lead the European Federation of Neurological Societies (EFNS) to advise that indications for DBS in CPSP are ‘equivocal’ and that further comparative trials are necessary [14] a recommendation that we fully endorse.

In conclusion we report a case of refractory CPSP due to a large temporo-parietal infarct that was improved by DBS of the PVG. This improvement was associated with a normalisation of hemi-body sensory inattention. Although we cannot prove causation, we speculate that the beneficial action of DBS of the PVG in this case is via restoration of normal sensory transmission of innocuous exteroceptive stimuli to higher centres perhaps by an action on the thalamus. This holds out the promise that even in the case of extensive CNS damage the pain generating mechanisms may be amenable to neuromodulatory approaches such as DBS.

## Figures and Tables

**Table 1:** Neuropathy pain scale. Scores for each individual aspect of pain (out of 10) assessed before the insertion of deep brain stimulator and measured again 6 weeks later.

Neuropathy pain scale	Before DBS	After DBS
Intensity	10	7
Sharp	10	7
Hot	2	0
Dull	10	6
Cold	10	10
Sensitive	10	5
Itchy	0	0
Unpleasant	10	7
Deep	10	0
Surface	10	7
